# Annual stability of the plasma Aß40/42 ratio and associated factors

**DOI:** 10.1002/acn3.51770

**Published:** 2023-04-04

**Authors:** Takumi Nakamura, Takeshi Kawarabayashi, Naoko Nakahata, Ken Itoh, Kazushige Ihara, Shigeyuki Nakaji, Yoshio Ikeda, Masamitsu Takatama, Mikio Shoji

**Affiliations:** ^1^ Department of Neurology Gunma University Graduate School of Medicine 3‐39‐22 Showa‐machi Maebashi 371‐8511 Japan; ^2^ Department of Social Medicine Hirosaki University Graduate School of Medicine 5 Zaifu‐cho Hirosaki 037‐8562 Japan; ^3^ Geriatrics Research Institute and Hospital 3‐26‐8 Otomo‐machi Maebashi 371‐0847 Japan; ^4^ Department of Rehabilitation Sciences, Division of Speech‐Language‐Hearing Therapy, School of Health Sciences Hirosaki University of Health and Welfare Hirosaki Aomori 036‐8102 Japan; ^5^ Department of Stress Response Science Hirosaki University Graduate School of Medicine 5 Zaifu‐cho Hirosaki 037‐8562 Japan

## Abstract

**Objective:**

The plasma Aß40/42 ratio is a biomarker of brain amyloidosis. However, the threshold difference between amyloid positivity and negativity is only 10–20% and fluctuates with circadian rhythms, aging, and *APOE‐ε4* during the decades of evolution of Alzheimer's disease.

**Methods:**

Plasma Aß40 and Aß42 levels in 1472 participants aged between 19 and 93 years in the Iwaki Health Promotion Project for 4 years were statistically analyzed.

**Results:**

The means and standard deviations of annual inter‐individual coefficients of variation were 5.3 ± 3.2% for Aß40, 7.8 ± 4.6% for Aß42, and 6.4 ± 4.1% for the Aß40/42 ratio. No significant age‐dependent changes were observed in inter‐individual coefficients of variation. Age‐dependent increases in Aβ42 levels were suppressed, whereas those in the Aβ40/42 ratio were enhanced in *APOE‐ε4* carriers. The change points of Aß42, Aß40, and the Aß40/42 ratio were 36.4, 38.2, and 43.5 years, respectively. In the presence of *APOE‐ε4*, the Aß40/42 ratio increased in middle‐aged and elderly subjects while Aβ42 levels decreased in elderly subjects.

**Interpretation:**

Individual values for Aß40, Aß42, and the Aß40/42 ratio did not fluctuate annually or in an age‐dependent manner. If the plasma Aβ40/42 ratio changes by more than 14.7% (+2 standard deviations) relative to age‐ and *APOE‐ε4*‐adjusted normal annual fluctuations, other biomarkers also need to be examined.

## Introduction

Alzheimer's disease (AD) is the most common cause of dementia and is characterized by the accumulation of amyloid‐beta (Aβ) and phosphorylated tau, which leads to neurodegeneration in the brain.^1^ In the AD continuum, the deposition of Aß is initiated 20 years before the onset of symptomatic cognitive decline, which, in turn, induces the production of phosphorylated tau. After a long incubation period of disease processes, neurofibrillary tangles and neurodegeneration emerge and cognitive decline advances during a decade of clinical dementia.^2^, ^3^, ^4^ The natural course of AD was proposed by a detailed study of the dominantly inherited Alzheimer Network^2^ and has been endorsed in sporadic AD cohort studies.^5^, ^6^, ^7^, ^8^ The signatures of these pathological and clinical processes may now be traced using biomarkers in cerebrospinal fluid and plasma Aß, phosphorylated tau, and neurofilament light chains as well as positron emission tomography on Aß and tau.^1^ Among these biomarkers of AD, the plasma Aß42/40 ratio has been established as a biomarker for Aß brain amyloidosis.^9^, ^10^, ^11^, ^12^ Therefore, measurements of the plasma Aß42/40 ratio have recently been attracting increasing attention as a non‐invasive and low‐cost screening method for brain amyloidosis in more precise examinations and for monitoring the efficacies of disease‐modifying therapies for AD. Aß amyloidosis consistently progresses in the 4 decades from the initial deposition of Aß to the end stage of dementia in AD. Although a cross‐sectional analysis of the plasma Aß ratio clearly showed significant differences between AD and controls, the difference in these values was expected to be very small (10–15%)^13^ during an extremely long disease duration. Plasma Aβ concentrations are affected by diurnal variations,^14^ aging,^15^ the presence of *APOE‐ε4*,^15^ and measurement methods.^16^, ^17^ Therefore, longitudinal studies on changes in the plasma Aß ratio and the identification of related factors are needed to establish biomarkers of Aß amyloidosis.

The present study investigated (1) annual fluctuations in individual plasma levels of Aß40 and Aß42 and the Aß40/42 ratio, (2) the effects of aging and the presence of *APOE‐ε4* on longitudinal plasma Aβ levels, and (3) the relationship between blood test parameters and plasma Aß species in the Iwaki Health Promotion Project.^18^

## Methods

### Participants

**Figure 1:**
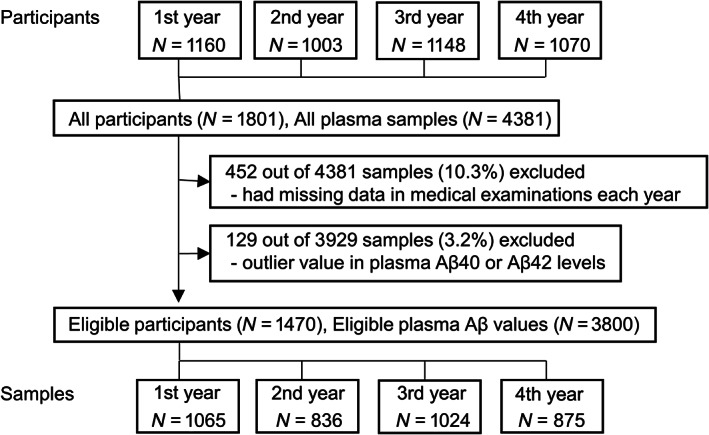
Flow diagram of cohort participants. *N*, number.

Subjects who participated in the Iwaki Health Promotion Project for 4 years between 2014 and 2017 were included in the present study. This project is a large longitudinal regional cohort study in the Iwaki area of Aomori Prefecture, Japan, in which participants undergo a series of medical checks each year, including cognitive tests, exercise capacity, blood pressure, height, weight, body fat percentage, a complete blood count, liver function, renal function, diabetes markers, lipid metabolism and endocrinology markers, immunological markers, cardiovascular biomarkers, and a urinalysis with 3000 items.^15^, ^18^, ^19^ A total of 1801 participants who were followed up at least once during annual medical check‐ups over a 4‐year period were eligible for subsequent analyses. Aβ40 and Aß42 levels were measured in 4381 plasma samples. Of these, 452 samples with missing data and 129 samples with outliers for Aβ40 or Aβ42 levels were excluded. Therefore, 1470 participants (3800 plasma samples) were included in the statistical analysis (Fig. 1 and Table 1). Written informed consent for medical examinations, including genetic testing, was obtained from all participants.

**Table 1:** Characteristics of eligible participants and plasma samples.

Characteristics	1st year	2nd year	3rd year	4th year	Eligible participants
Number	1065	836	1024	875	1470 (3800 samples)
Age	54.3 (15.3)	55.2 (14.6)	53.9 (15.6)	55.4 (14.8)	54.6 (15.1)
Sex (male/female)	403/662	305/531	400/624	355/520	573/897
MMSE scores	29.3 (1.3)	29.3 (1.3)	29.3 (1.4)	29.4 (1.2)	29.3 (1.3)
Renal impairment moderate/severe (*N* (%))	84 (7.9)/1 (0.0)	68 (8.1)/1 (0.0)	84 (8.2)/0 (0.0)	82 (9.3)/0 (0.0)	
Hypertension (*N* (%))	346 (32.5)	161 (19.3)	251 (24.5)	166 (18.9)	
Overweight (*N* (%))	223 (20.9)	196 (23.4)	247 (24.1)	215 (24.5)	
Dyslipidemia (*N* (%))	303 (28.5)	266 (31.8)	334 (32.6)	279 (31.9)	
Diabetes (*N* (%))	10 (0.9)	8 (1.0)	34 (3.3)	26 (3.0)	
*APOE* genotype (number)					
*APOE‐ε2/ε2*	0	1	2	1	2
*APOE‐ε2/ε3*	72	50	73	60	101
*APOE‐ε3/ε3*	772	609	730	629	1060
*APOE‐ε2/ε4*	11	7	7	7	12
*APOE‐ε3/ε4*	204	161	200	170	282
*APOE‐ε4/ε4*	6	8	12	8	13
Aβ levels					
Aβ40 (pmol/mL)	105.5 (15.5)	100.1 (15.3)	102.6 (15.2)	100.0 (15.4)	102.3 (15.5)
Aβ42 (pmol/mL)	11.4 (1.7)	10.9 (1.7)	12.3 (2.1)	11.7 (2.0)	11.6 (2.0)
Aβ40/42 ratio	9.3 (1.1)	9.3 (1.0)	8.4 (1.2)	8.6 (1.0)	8.9 (1.1)

MMSE, mini‐mental state examination; Aβ, amyloid‐beta.

Age: mean and standard deviation; Sex: number; MMSE scores: mean and standard deviation; Aß levels: mean and standard deviation.

Moderate renal impairment was defined as eGFR ≥30 mL/min/m^2^ and eGFR <60 mL/min/m^2^; severe renal impairment was defined as eGFR <30 mL/min/m^2^; hypertension was defined as systolic blood pressure ≥140 mmHg or diastolic blood pressure ≥ 90 mmHg; overweight was defined as body mass index ≥25; dyslipidemia was defined as low‐density lipoprotein cholesterol ≥140 mg/dL, high‐density lipoprotein cholesterol <40 mg/dL, or triglycerides ≥150 mg/dL; diabetes was defined as blood sugar ≥126 mg/dL and hemoglobin A1c ≥6.5%.

### Plasma Aβ measurements

Ten milliliters of morning fasting blood was collected into an EDTA‐2Na tube and immediately centrifuged at 1400× *g* for 10 min, separated into plasma in a polypropylene tube, and stored at −80°C for later analyses. Sandwich ELISA was used to quantify plasma Aβ40 and Aβ42 levels using the Human/Rat ß Amyloid (40) ELISA Kit Wako II and Human/Rat ß Amyloid (42) ELISA Kit Wako High‐Sensitive (Wako Pure Chemical Industries, Ltd., Osaka, Japan). The antibodies used and assay sensitivities were previously described.^15^, ^20^, ^21^, ^22^ The 129 outliers of plasma Aβ40 or Aβ42 levels were excluded by the ROUT method (*Q* = 1%).^23^

### *APOE* genotyping

The DNA of Iwaki residents was purified from peripheral whole blood using the QIAamp® 96 DNA Blood Kit (QIAGEN, Hilden, Germany), and the *APOE* genotype was identified by Toshiba Corporation using the Japonica Array consisting of population‐specific SNP markers designed from the 1070 whole genome reference panel. The primers used were previously reported.^15^, ^18^

### Statistical analysis

Plasma Aβ40 and Aβ42 values and the Aβ40/42 ratio did not significantly deviate from the normal distribution by histograms and QQ plots. To assess the extent to which plasma Aβ levels fluctuated among repeated annual measurements, the coefficient of variation (CV) was calculated within individuals who underwent measurements multiple times and was plotted in histograms. Individual CV values at the mean age during the interval of repeated measurements were analyzed by a regression analysis.

Linear mixed‐effects models were used in the data analysis of *APOE* allele‐dependent variations in plasma Aβ levels. Models were created that included plasma Aβ species as the dependent variable, age and the estimated glomerular filtration rate (eGFR) as fixed effects,^15^ and repeated subjects (intercept and slope) as random effects.

A change point analysis was performed on these models to identify changes in the linear relationship between plasma Aβ levels and aging. The change points of plasma Aβ40 and Aβ42 levels and the Aβ40/42 ratio obtained from these analyses were used in a mixed‐effect segmented regression model.^8^

To clarify whether age‐dependent changes in plasma Aβ species were affected by the *APOE* allele, the *APOE‐ε2* or *APOE‐ε4* allele and the interaction between these alleles and age were added to the model as fixed effects. We conducted a comparative analysis of plasma Aβ levels in individuals with diverse *APOE* genotypes, including the presence of *APOE‐ε2* and *APOE‐ε4* alleles, as well as the co‐occurrence of *APOE‐ε3* and examined the potential for Aβ variations across the different genotypes. To analyze the variability of plasma Aβ levels according to each *APOE* genotype, we initially averaged plasma Aβ levels measured multiple times for each subject. Subjects were then divided into the following age quartiles: Group 1: ≤40 years, Group 2: 41–55 years of age, and Group 3: ≥56 years. Groups were stratified based on the change point of plasma Aβ, as selected by a prior statistical analysis, and the age at which the onset of a decline in the mini‐mental state examination (MMSE) manifested in this cohort. Plasma Aβ values between each *APOE* genotype were compared by a one‐way analysis of variance, and Tukey's multiple comparison post hoc test was performed for each age group.

To examine the relationships between plasma Aβ levels and laboratory values, values were averaged for each subject. Regarding laboratory values that did not follow a normal distribution, a log transformation (white blood cell count, bilirubin, aspartate aminotransferase, alanine aminotransferase, γ‐glutamyl transpeptidase, C‐peptide, and brain natriuretic peptide) or Box‐Cox transformation (ferritin, free thyroxine, and glycoalbumin) was applied. A multiple regression analysis was then conducted with plasma Aβ levels as the dependent variable and laboratory values as the independent variable adjusted for age.

In this cohort population, MMSE scores began to decline at 55 years.^18^ A score of 23/24 is the widely used cutoff value for MMSE.^24^ Therefore, to evaluate the relationship between plasma Aβ species and MMSE, we initially divided subjects aged ≥55 years into two groups: one group with an MMSE score ≤ 23 at least once and one group that maintained an MMSE score ≥ 24. In these two groups, we created a binomial logistic regression adjusted for age, the presence of *APOE‐ε4*, and grouped educational history (≤9 years, 10–15 years, and ≥16 years), and compared plasma Aβ levels averaged within individuals.

All tests were two‐tailed, and significance was set at 5%. GraphPad Prism version 9 (GraphPad Software, San Diego, CA) was used to exclude outliers using the ROUT method. R version 3.5.1 was used for other statistics. The package lme4 version 1.1.19 was employed for mixed‐effects models, lmerTest version 3.1.0 was applied to calculate p‐values for mixed‐effects models, and MASS version 7.3.51.1 was used for the one‐way analysis of variance.

## Results

### Annual stability of plasma Aß levels and age dependency

**Figure 2:**
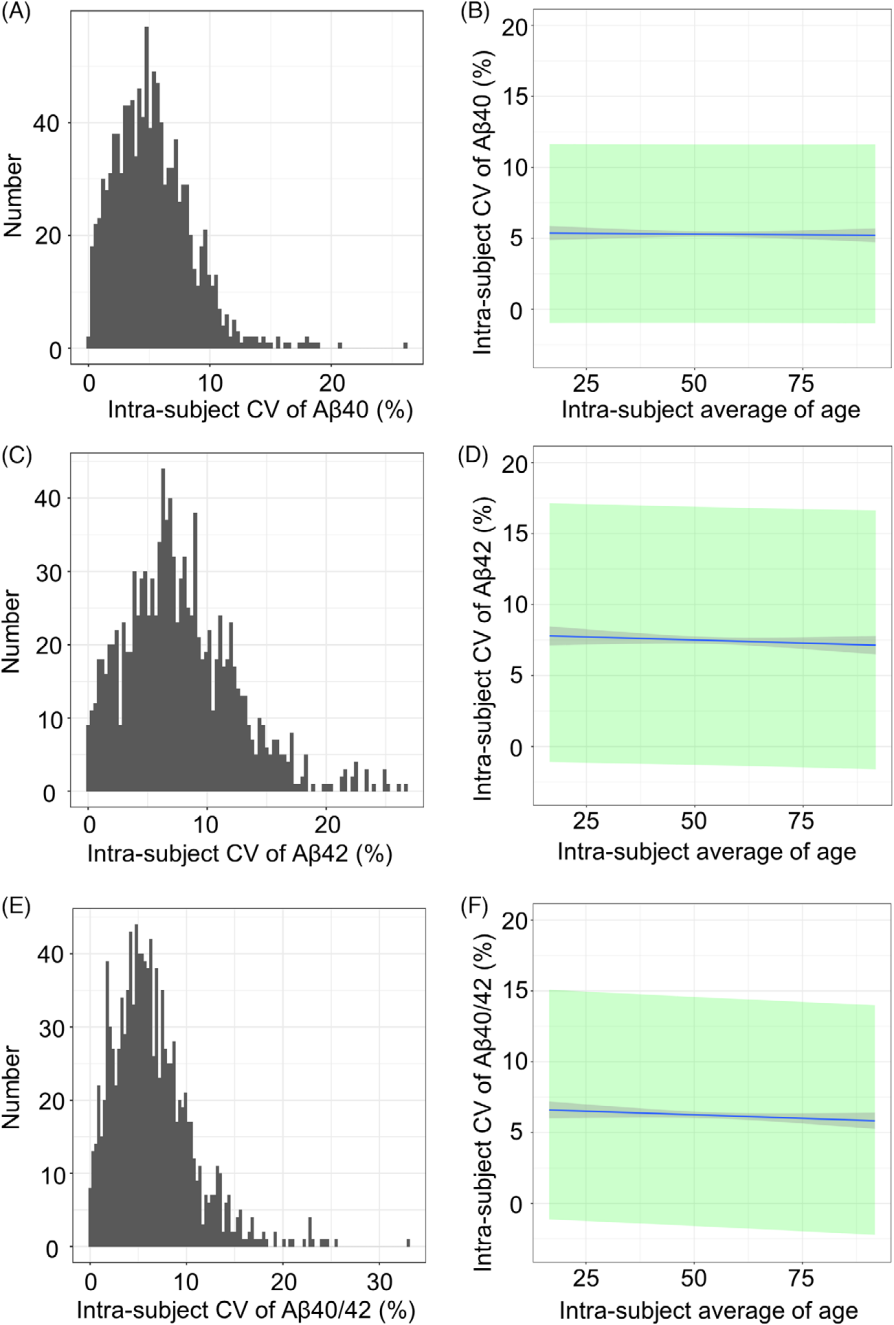
Inter‐subject CV of longitudinal measurements of plasma Aβ. CV, coefficient of variation; Aβ, amyloid‐beta. Distribution histograms of values for the coefficient of variations (CV) of Aβ40 levels (A), Aβ42 levels (C), and the Aβ40/42 ratio (E). The regression lines between the intra‐subject average of age and intra‐subject CV of Aβ (B), Aβ42 (D), and the Aβ40/42 ratio (F). The gray area indicates the 95% confidence interval (CI) of regression lines, while the green area shows the 95% prediction interval. The means and SD of CV were 5.32 ± 3.20% (95% CI: 5.13–5.51%) for Aß40, 7.78 ± 4.63% (95% CI: 7.51–8.05%) for Aß42, and 6.43 ± 4.12% (95% CI: 6.19–6.67%) for the Aß40/42 ratio. The 2SD upper limits of individual annual CVs were 11.71% for Aß40, 17.04% for Aß42, and 14.67% for the Aß40/42 ratio. No significant age‐dependent changes were observed in Aβ40 (Fig. 2B, regression equation; *Y* = −0.001*X* + 5.35, coefficient of determination; *r* ^2^ < 0.001, *P* = 0.930), Aβ42 (Fig. 2D, *Y* = −0.008*X* + 8.19, *r* ^2^ < 0.001, *P* = 0.407), or the Aβ40/42 ratio (Fig. 2F, *Y* = −0.02X + 7.25, *r* ^2^ = 0.003, *P* = 0.066).

The inter‐individual CV of repeated annual measurements of Aß40, Aß42, and the Aß40/42 ratio were plotted in histograms (Fig. 2A, C and E). The means and SD of CV were 5.32 ± 3.20% (95% confidence interval (CI): 5.13–5.51%) for Aß40, 7.78 ± 4.63% (95% CI: 7.51–8.05%) for Aß42, and 6.43 ± 4.12% (95% CI: 6.19–6.67%) for the Aß40/42 ratio. The 2SD upper limits of the annual inter‐individual CV were 11.71% for Aß40, 17.04% for Aß42, and 14.67% for the Aß40/42 ratio. No significant age‐dependent changes in mean CV were observed in Aβ40 (Fig. 2B, regression equation; *Y* = −0.001*X* + 5.35, coefficient of determination; *r* ^2^ < 0.001, *P* = 0.930), Aβ42 (Fig. 2D, *Y* = −0.008*X* + 8.19, *r* ^2^ < 0.001, *P* = 0.407), or the Aβ40/42 ratio (Fig. 2F, *Y* = −0.02*X* + 7.25, *r* ^2^ = 0.003, *P* = 0.066). These results suggest that the individual values for Aß40, Aß42, and the Aß ratio did not so fluctuate annually, and the fluctuation level did not change with aging.

### Effects of the *APOE* allele on age‐dependent changes in plasma Aβ levels

The results of linear mixed‐effects models are shown in Table 2. In linear mixed‐effect models including *APOE‐ε4*, Aβ40 levels increased with aging, and this was not affected by the presence of *APOE‐ε4*. Aβ42 levels increased with aging, and *APOE‐ε4* suppressed these age‐dependent increases. The Aβ40/42 ratio increased with aging, and *APOE‐ε4* enhanced this age‐dependent increase. In mixed‐effect models including the presence of *APOE‐ε2*, values for Aβ40, Aβ42, and the Aβ40/42 ratio increased with aging, and age‐dependent increases did not vary with the presence of *APOE‐ε2*. As a result, the age‐dependent increase in Aβ42 levels was suppressed, whereas that in the Aβ40/42 ratio was enhanced in *APOE‐ε4* carriers. Age‐dependent Aβ increases were not affected by the presence of *APOE‐ε2*.

**Table 2:** Results of linear mixed‐effects models including *APOE*.

Aβ species	Aβ40				Aβ42				Aβ40/42 ratio			
Linear mixed‐effects models including *APOE*												
Model includes	*APOE‐ε4*		*APOE‐ε2*		*APOE‐ε4*		*APOE‐ε2*		*APOE‐ε4*		*APOE‐ε2*	
Fixed effect	*β* (SE)	*P*	*β* (SE)	*P*	*β* (SE)	*P*	*β* (SE)	*P*	*β* (SE)	*P*	*β* (SE)	*P*
Intercept	101.10 (2.45)	<0.001	101.15 (2.38)	<0.001	13.14 (0.35)	<0.001	13.19 (0.34)	<0.001	7.67 (0.19)	<0.001	7.60 (0.18)	<0.001
Age	0.35 (0.03)	<0.001	0.34 (0.02)	<0.001	0.02 (0.004)	<0.001	0.01 (0.003)	>0.001	0.02 (0.002)	<0.001	0.02 (0.002)	<0.001
*APOE*	−0.12 (2.88)	0.966	−2.45 (4.26)	0.566	0.39 (0.38)	0.312	−0.13 (0.57)	0.826	−0.40 (0.19)	0.037	−0.03 (0.29)	0.913
eGFR	−0.22 (0.02)	<0.001	−0.22 (0.02)	<0.001	−0.03 (0.003)	<0.001	−0.03 (0.003)	<0.001	0.002 (0.001)	0.194	0.002 (0.001)	0.163
Age × *APOE*	−0.002 (0.05)	0.977	0.08 (0.08)	0.3041	−0.02 (0.01)	0.028	0.01 (0.01)	0.365	0.01 (0.004)	<0.001	−0.002 (0.01)	0.752

Aβ, amyloid‐beta; SE, standard error; eGFR, estimated glomerular filtration rate.

*β* is the fixed effect and *P* is the *P*‐value. “×” indicates an interaction between two variables.

### Change point analysis of plasma Aß species

**Figure 3:**
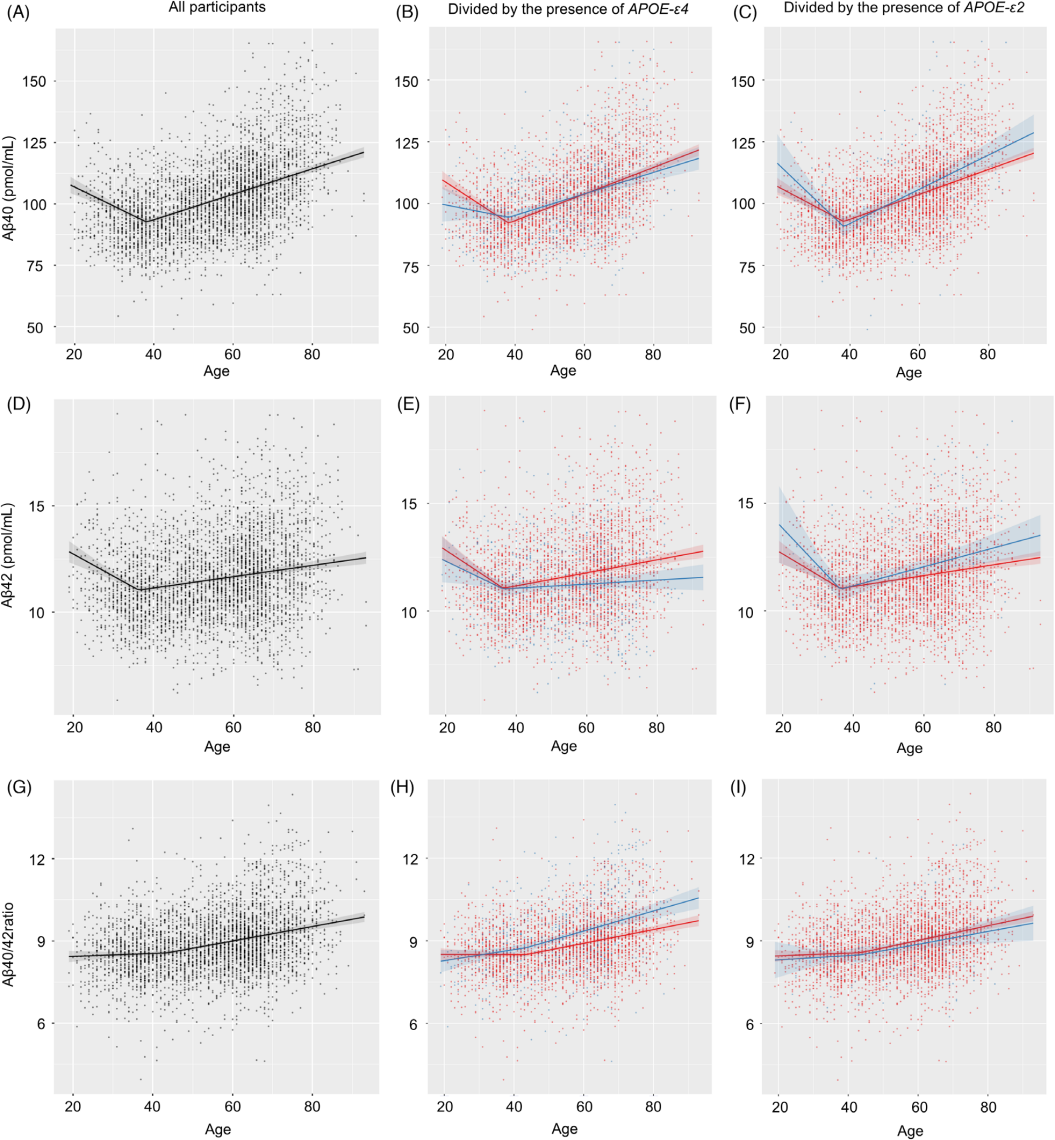
Change point analysis of Aß levels in the presence of *APOE‐ε4* and *APOE‐ε2*. Black plots and lines indicate all subjects. Blue indicates *APOE‐ε4* or *APOE‐ε2* carriers, while red shows the corresponding non‐carriers. All samples for the entire year are plotted. Lines indicate regression lines and the 95% confidence intervals of regression lines. Plasma Aβ40 levels decreased until 38.2 years and then increased (A). Plasma Aβ42 levels decreased until 36.4 years and then increased (D). The plasma Aβ40/42 ratio increased until 43.5 years and then showed enhanced age‐dependent increases (G). Decreases in Aβ40 levels with aging were attenuated in the presence of *APOE‐ε4* until 38.2 years (B). Aβ42 levels were not affected by *APOE‐ε4* before or after 36.4 years (E). The increase in the Aβ40/42 ratio after 43.5 years was enhanced in the presence of *APOE‐ε4* (H). *APOE‐ε2* did not affect age‐dependent changes in Aβ40, Aβ42, or the Aβ40/42 ratio before or after each change point (C, F, I).

We examined change points in age‐ and *APOE‐ε4*‐dependent increases in Aß. The change point of Aß40 levels was 38.2 years. The former fixed effect of the change point was −0.79, while the latter was 0.52 (Fig. 3A). Regarding Aß42 levels, the change point was 36.4 years. The former fixed effect was −0.10, while the latter was 0.03 (Fig. 3D). The change point of the Aß40/42 ratio was 43.5 years, and the former and latter fixed effects were 0.01 and 0.03 (Fig. 3G), respectively. In both models, Akaike's information criterion improved when change points were included (Aβ40: from 28948.88 to 28820.54; Aβ42: from 14690.01 to 14633.85; and Aβ40/42 ratio: from 10816.86 to 10802.36). The results of the change point analysis are shown in the upper part of Table 3. Therefore, Aß40 and Aß42 levels decreased in subjects until their late 30s. After this point, Aß42 and Aß40 levels continuously increased. Increases in the Aß40/42 ratio were initially detected at 41 years.

**Table 3:** Results of the change point analysis and mixed‐effects models including change points and *APOE*.

Aβ species	Aβ40	Aβ42	Aβ40/42 ratio
CP (95% CI)	38.2 (36.7–39.6)	36.4 (34.1–38.6)	43.5 (36.3–50.7)
Former/latter slope of CP (95% CI)	−0.79 (−1.02 to −0.55)/0.52 (0.46 to 0.58)	−0.10 (−0.14 to −0.06)/0.03 (0.02 to 0.03)	0.01 (−0.07 to 0.02)/0.03 (0.02 to 0.03)

Mixed effect models including change points and *APOE*												
Model includes	*APOE‐ε4*		*APOE‐ε2*		*APOE‐ε4*		*APOE‐ε2*		*APOE‐ε4*		*APOE‐ε2*	
Fixed effect	*β* (SE)	*P*	*β* (SE)	*P*	*β* (SE)	*P*	*β* (SE)	*P*	*β* (SE)	*P*	*β* (SE)	*P*
Intercept	128.93 (2.69)	<0.001	126.32 (2.60)	<0.001	15.40 (0.40)	<0.001	15.21 (0.38)	<0.001	8.41 (0.19)	<0.001	8.33 (0.19)	<0.001
Age (former CP)	−17.49 (2.10)	<0.001	−14.26 (1.95)	<0.001	−1.88 (0.31)	<0.001	−1.71 (0.29)	<0.001	−0.01 (0.14)	0.96	0.12 (0.13)	0.344
Age (latter CP)	12.09 (2.10)	<0.001	13.42 (1.98)	<0.001	−0.16 (0.31)	0.614	−0.27 (0.29)	0.358	1.23 (0.14)	<0.001	1.44 (0.13)	<0.001
*APOE*	−9.85 (3.98)	0.013	9.36 (6.18)	0.13	−0.54 (0.60)	0.37	1.27 (0.94)	0.177	−0.25 (0.23)	0.287	−0.15 (0.35)	0.669
eGFR	−0.24 (0.02)	<0.001	−0.24 (0.02)	<0.001	−0.03 (0.003)	<0.001	−0.03 (0.003)	<0.001	0.001 (0.002)	0.425	0.002 (0.002)	0.339
Age × *APOE* (former CP)	12.20 (4.57)	0.008	−11.40 (7.29)	0.118	0.51 (0.68)	0.455	−1.30 (1.08)	0.228	0.50 (0.28)	0.08	0.07 (0.44)	0.878
Age × *APOE* (latter CP)	6.47 (4.32)	0.134	−1.08 (6.47)	0.868	−0.68 (0.63)	0.286	−0.23 (0.96)	0.809	1.07 (0.28)	<0.001	−0.10 (0.41)	0.801

Aβ, amyloid‐beta; SE, standard error; CP, change point; eGFR, estimated glomerular filtration rate.

### Effects of *APOE* on plasma Aß levels before or after change points

*APOE‐ε4* suppressed age‐dependent decreases in Aß40 levels before the change point, but showed no differences thereafter (Fig. 3B). In the presence of *APOE‐ε2*, no significant age‐dependent differences were observed before and after (Fig. 3C) change points. The presence of *APOE‐ε4* or *APOE‐ε2* did not significantly change age‐dependent decreases or increases in Aβ42 before or after change points (Fig. 3E and F).

Age‐dependent increases in the Aβ40/42 ratio were unaffected before the change point; however, the increase in the Aß40/42 ratio was emphasized after change points in the presence of *APOE‐ε4* (Fig. 3H). In the presence of *APOE‐ε2*, no significant differences were observed in age‐dependent increases in the Aβ40/42 ratio before and after change points (Fig. 3I). The results of mixed‐effect models including change points and *APOE‐ε2* or *ε4* are shown in the lower part of Table 3. Therefore, the Aß40/42 ratio increased further in an age‐dependent manner in the presence of *APOE‐ε4* from middle age. *APOE‐ε2* did not affect age‐dependent changes in Aß levels before and after the change points.

### Age‐dependent changes in plasma Aß levels in each *APOE* genotype

**Figure 4:**
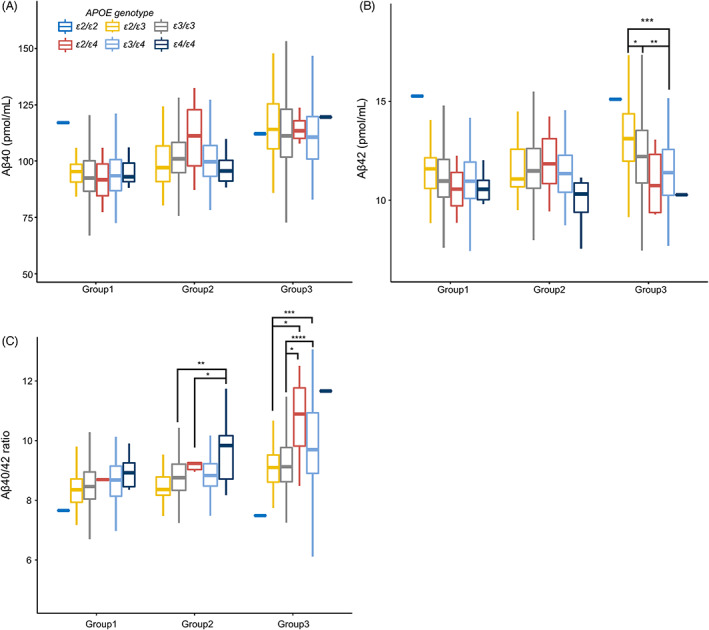
Age‐dependent changes in plasma Aß levels according to *APOE* genotypes. One‐way analysis of variance models for comparisons of plasma Aβ levels with *APOE* genotypes between different generations. Aβ40 levels were not affected by the *APOE* genotype in either generation. Aβ42 levels in the elderly group significantly differed in the *APOE‐ε2/ε3* > *APOE‐ε3/ε3*, *APOE‐ε2/ε3* > *APOE‐ε3/ε4*, and *APOE‐ε3/ε3* > *APOE‐ε3/ε4* groups. The Aβ40/42 ratio in the middle‐aged group significantly differed in the *APOE‐ε3/ε3* > *APOE‐ε4/ε4* and *APOE‐ε2/ε4* > *APOE‐ε4/ε4* groups. The Aβ40/42 ratio in the elderly group was high in the order of *APOE‐ε2/ε3* = *APOE‐ε3/ε3* > *APOE‐ε2/ε4* = *APOE‐ε3/ε4*.

There were no *APOE‐ε2/ε2* subjects in the middle‐aged group, only one *APOE‐ε2/ε2* subject in the young group, and one *APOE‐ε2/ε2* and one *APOE‐ε4/ε4* subject each in the elderly group; therefore, they were excluded from multiple comparisons. No significant differences were observed in Aß40 levels among the young (p = 0.968), middle‐aged (*P* = 0.073), and elderly (*P* = 0.182) groups for every *APOE* genotype (Fig. 4A). Furthermore, no significant differences were noted in Aβ42 levels among the young (*P* = 0.573) and middle‐aged (*P* = 0.058) groups, whereas significant differences were detected in the elderly group (*P* < 0.001). In multiple comparisons, Aβ42 levels in the elderly group were significantly different from those in the *APOE‐ε2/ε3* > *APOE‐ε3/ε3* (mean difference 0.88, 95% CI: 0–1.76, *P* = 0.048), *APOE‐ε2/ε3* > *APOE‐ε3/ε4* (mean difference 1.71, 95% CI: 0.72–2.70, *P* < 0.001), and *APOE‐ε3/ε3* > *APOE‐ε3/ε4* (mean difference 0.83, 95% CI: 0.25–1.41, *P* = 0.002) groups (Fig. 4B). Regarding the Aβ40/42 ratio, significant differences were noted in the middle‐aged (*P* = 0.001) and elderly (*P* < 0.001) groups, but not in the young group (*P* = 0.177). Multiple comparisons of the Aβ40/42 ratio in the middle‐aged group showed significant differences in the *APOE‐ε4/ε4* > *APOE‐ε3/ε3* (mean difference 1.10, 95% CI: 0.24–1.95, *P* = 0.004) and *APOE‐ε4/ε4* > *APOE‐ε2/ε4* (mean difference 0.87, 95% CI: 0.09–1.65, *P* = 0.002) groups. In the elderly group, multiple comparisons of the Aβ40/42 ratio showed significant differences in *APOE‐ε2/ε4* > *APOE‐ε2/ε3* (mean difference 1.58, 95% CI: 0.11–3.05, *P* = 0.03), *APOE‐ε3/ε4* > *APOE‐ε2/ε3* (mean difference 0.84, 95% CI: 0.28–1.41, *P* = 0.001), *APOE‐ε2/ε4* > *APOE‐ε3/ε3* (mean difference 1.41, 95% CI: 0.01–2.81, p = 0.048), and *APOE‐Εε3/ε4* > *APOE‐ε3/ε3* (mean difference 0.67, 95% CI: 0.34–1.00, *P* < 0.001) (Fig. 4C). Therefore, *APOE‐ε4* decreased Aβ42 levels and increased the Aβ40/42 ratio after middle age. The Aβ40/42 ratio was affected early in life.

### Relationships among blood test data, Aβ 40, Aß42, and the Aß40/42 ratio

Aβ40, Aβ42, and the Aβ40/42 ratio correlated with hemoglobin, alanine aminotransferase, γ‐glutamyl transpeptidase, ferritin, and glycoalbumin. Bilirubin, aspartate aminotransferase, albumin, eGFR, potassium, insulin, C‐peptide, and brain natriuretic peptide correlated with Aβ40 and Aβ42 levels, but not with the Aβ ratio. Triglycerides and blood glucose only correlated with the Aβ ratio. The white blood cell count, uric acid, total cholesterol, high‐density lipoprotein cholesterol, low‐density lipoprotein cholesterol, and free thyroxine correlated with Aβ40 levels and the Aβ ratio (Table 4).

**Table 4:** Relationships between laboratory data and plasma Aβ levels.

	Aβ40			Aβ42			Aβ40/42 ratio		
	*P*	*β*	*R*	*P*	*β*	*R*	*P*	*β*	*R*
WBC	<0.001	17.02	0.30	0.228	0.48	0.06	<0.001	1.03	0.16
Hb	<0.001	−0.99	0.29	<0.001	−0.20	0.08	<0.001	0.07	0.15
Bilirubin	0.010	−1.87	0.28	0.020	−0.22	0.06	0.722	0.02	0.14
AST	0.013	−6.73	0.28	<0.001	−1.20	0.06	0.061	0.36	0.14
ALT	<0.001	−6.58	0.29	<0.001	−1.16	0.07	0.008	0.31	0.15
γ‐GTP	<0.001	−4.07	0.29	<0.001	−0.90	0.08	<0.001	0.33	0.15
Albumin	0.005	−3.84	0.28	<0.001	−0.59	0.06	0.146	0.14	0.14
eGFR	<0.001	−0.29	0.34	<0.001	−0.03	0.11	0.942	0	0.14
UA	<0.001	0.95	0.29	0.420	−0.03	0.06	<0.001	0.09	0.16
TC	<0.001	−0.06	0.29	0.075	−0.002	0.06	<0.001	−0.003	0.15
TG	0.062	0.01	0.28	0.825	0	0.06	0.009	0.001	0.15
HDL	<0.001	−0.09	0.29	0.385	−0.002	0.06	<0.001	−0.01	0.15
LDL	<0.001	−0.05	0.29	0.094	−0.003	0.06	0.026	−0.002	0.15
Potassium	<0.001	4.43	0.29	<0.001	0.64	0.07	0.213	−0.11	0.14
BG	0.919	0.002	0.28	0.063	0.01	0.06	0.010	−0.004	0.15
Ferritin	0.002	−0.37	0.28	<0.001	−0.09	0.08	<0.001	0.04	0.15
fT4	0.009	6.17	0.28	0.860	−0.06	0.06	<0.001	0.58	0.15
Insulin	0.045	0.15	0.28	0.048	0.02	0.06	0.527	−0.003	0.14
C‐peptide	<0.001	8.28	0.29	0.041	0.60	0.06	0.181	0.21	0.14
GA	0.006	40.71	0.28	<0.001	7.51	0.06	0.020	−2.50	0.15
BNP	<0.001	4.00	0.29	<0.001	0.49	0.06	0.653	−0.04	0.14

WBC, white blood cell count; Hb, hemoglobin; AST, aspartate aminotransferase; ALT, alanine aminotransferase; γ‐GTP, γ‐glutamyl transpeptidase; eGFR, estimated glomerular filtration rate; UA, uric acid; TC, total cholesterol; TG, triglycerides; HDL, high‐density lipoprotein cholesterol; LDL, low‐density lipoprotein cholesterol; BG, blood glucose; fT4, free thyroxine; GA, glycoalbumin; BNP, brain natriuretic peptide.

Results of a multiple regression analysis with Aβ as the dependent variable and age and each parameter as explanatory variables. *P*‐values for each parameter were corrected for age. *β*‐values indicate standardized partial regression coefficients for each parameter. *R* indicates the multiple correlation coefficient in the multiple regression equation.

### The relationship between cognitive function and plasma Aβ

In the multivariate logistic regression between two MMSE groups with cutoffs of 23/24, the adjusted p‐values for Aβ40, Aβ42, and the Aβ40/42 ratio were 0.907, 0.431, and 0.433, respectively. Aging and a low level of educational history correlated with low MMSE scores in each model (all *P*‐values < 0.001). In these models, the presence of *APOE‐ε4* correlated with MMSE scores in the models of Aβ42 (p‐value 0.045, odds ratio 2.75, 95% CI 0.97–7.25) and Aβ40/42 (p‐value 0.039, odds ratio 2.96, 95% CI 1.10–8.10), but not in that of Aβ40 (*P*‐value 0.058). As we previously reported, MMSE scores were strongly influenced by age and educational history in our cohort and less so by the presence of *APOE‐ε4* and plasma Aβ levels.^15^, ^18^ Therefore, the present results are consistent with our previous findings.

## Discussion

The present study revealed that the average CV of inter‐individual measurements over time were 5.32% for Aß40, 7.76% for Aß42, and 6.43% for the Aß40/42 ratio. The 2SD upper limits of CV were 11.71% for Aß40, 17.04% for Aß42, and 14.67% for the Aß40/42 ratio. The inter‐individual CV of the values for Aβ40, Aβ42, and the Aβ40/42 ratio did not change in an age‐dependent manner. These results indicate that individual values for plasma Aß40, Aß42, and the Aß ratio do not fluctuate annually and are stable regardless of aging. A number of factors have been proposed to contribute to fluctuations in plasma Aß levels. The diurnal circadian pattern varies in amplitude from 2% to 4.1% in Aß40 and from 3.2% to 7.6% in Aß42.^14^ Intra‐ and inter‐assay CV% were 1.6–4.8 in Aß1‐42 and 1.7–2.6 in Aß40 by EUROIMMUN ELISAs, and 4.3–8.6 in Aß42 and 2.2–6.0 in Aß40 by the Quanterix SIMOA assay.^16^ An immunoprecipitation‐LC–MS/MS assay (IP‐MS) using the antibody HJ 5.1 showed intra‐ and inter‐assay CV% of 2.5–8.4 and 3.1–9.5 in Aß1‐42, and 1.5–3 and 2.7–7.7 in Aß40, respectively.^25^ Pre‐analytical sample handling with appropriate sampling, preparation, storage, and freeze–thaw cycles are recommended to reduce intra‐assay variability to <10%.^26^, ^27^ Our ELISA for plasma Aß40 and Aß42 corresponded to intra‐and inter‐assay CV <9% to 10%.^15^, ^22^ Samples are strictly regulated in pre‐analytical handling, such as fasting morning sampling, separation, and storage.^15^ Since these quality controls of our assay are satisfactory, the annual stability of plasma Aß levels independent of age is acceptable. If the annual stability of plasma Aβ as observed in the present study was disrupted, the dynamics of amyloid may change. Therefore, in addition to setting cutoff values, we propose other biomarkers that need to be considered if the plasma Aβ40/42 ratio fluctuates by more than 14.7% (+2SD) relative to normal annual fluctuations adjusted for age and *APOE‐ε4*.

The global standardization of SIMOA and other ELISAs, LC–MS, and IP‐MS showed weak correlations for Aß42, while Aß40 correlations were stronger. The Aß40/42 ratio showed a correlation, although it was weaker than that of Aß40.^17^ Another comparison using BioFINDER and ADNI samples by 8 plasma Aß42/40 assays, including IP‐MS, an antibody‐free liquid MS, and other immunoassays, showed a higher area under the receiver operating curve value (AUC) of 0.86 to the moderate AUC of 0.64 to accurately separate Aß‐positive samples from Aß‐negative samples.^28^

The plasma Aß42/40 ratio has an AUC of 0.88–0.97 for discriminating between brain Aß‐positive and Aß‐negative samples.^9^, ^13^ A 3‐year follow‐up of mild cognitive impairment showed that a lower Aß1‐42 level and Aß42/40 ratio correlated with the conversion to dementia.^29^ Six independent cohorts using IP‐MS reported significant differences with AUC of 0.81.^30^ Recent class II evidence indicated high accuracy with AUC of 0.84–0.91.^10^, ^11^, ^12^ However, these studies also showed the extensive overlapping of individual measurements among cognitively normal, mild cognitive impairment, and AD patients.^31^ The plasma Aß42/40 ratio was only 10–20% lower in patients with AD than in controls.^13^ Differences in the threshold concentration between converters and non‐converters were only 2.1 pg/mL (6.5%) for Aß 1–42 and 0.006 (3.9%) for the Aß42/40 ratio.^29^ In the AIBL cohort analyzing cognitively normal controls older than 60 years, brain Aß deposits had increased by 8.8% annually for 17 years before mild AD. The plasma Aß42/40 ratio had increased by 7.9% annually for 5 years before mild AD. The trajectory for plasma amyloid proceeds that for brain amyloid by a median value of 6 years.^7^ Since brain amyloidosis appears and develops 2 decades before mild cognitive impairment, it is important to decide what percentage change in the Aß42/40 ratio is meaningful during long‐term screening periods. Therefore, our longitudinal data analyzing factors affecting plasma Aß levels proposes basic information to select cutoff values for longitudinal screening.

This is the first study to employ a change point analysis of plasma Aß. A cross‐sectional study of 3284 cognitively normal individuals aged between 18 and 101 years revealed accelerated changes in the cerebrospinal fluid Aß42/Aß40 ratio at 46 years, Aß42 at 48 years, and amyloid positron emission tomography at 54 years.^8^ The present cohort consisted of 94.1% cognitively healthy participants, 5.4% with mild cognitive impairment, and 0.5% with dementia, and MMSE scores began to decline after 55 years.^18^ In the present study, change points were 36.4 years for Aß42, 38.2 years for Aß40, and 43.5 years for the Aβ40/42 ratio. Changes in plasma Aβ42 levels began to appear approximately 19 years before the decline in MMSE scores in our cohort. Change points for the plasma Aß40/42 ratio emerged 5 years before that for the cerebrospinal fluid Aß42/40 ratio in a Washington cohort.^8^ Therefore, the natural course of changes in plasma Aß levels may be earlier than those in cerebrospinal fluid; however, assay differences may also have contributed to this discrepancy.

We previously showed that age‐dependent increases in plasma Aß42 levels were suppressed by the presence of *APOE‐ε4* and recommended adjustments for age and *APOE‐ε4* in evaluations of plasma Aß levels as biomarkers.^15^ The present longitudinal study supports further detailed adjustments. *APOE‐ε4* did not affect age‐dependent increases in Aβ40 levels, but suppressed those in Aβ42 levels and enhanced those in the Aβ40/42 ratio. *APOE‐ε4* attenuated decreases in Aβ40 levels with aging until the change point at 38.2 years, did not affect Aβ42 levels before or after 36.4 years, and increased the Aβ40/42 ratio after the change point of 43.5 years. *APOE‐ε2* did not affect age‐dependent changes in Aβ40, Aβ42, or the Aβ40/42 ratio before or after each change point. These results were also confirmed using one‐way analysis of variance models for comparisons of plasma Aβ levels among different generations according to *APOE* genotypes, namely *APOE‐ε4* significantly affected the Aß40/42 ratio from the middle‐aged group and Aß42 levels from the elderly group. As recently reported, plasma Aβ42/40 may be employed to predict amyloid positron emission tomography positivity, with the prediction accuracy being improved by the addition of the *APOE‐ε4* status.^10^, ^11^, ^12^ Enhancements in AUC combined with the *APOE‐ε4* status are considered to be associated with age and *APOE‐ε4*‐dependent changes in the plasma Aß40/42 ratio and Aß42 levels.

The present study showed that plasma Aβ40, Aβ42, and the Aβ40/42 ratio were associated with multiple blood chemistry items. However, no relationships were observed between the plasma Aβ40/42 ratio and kidney damage (a low eGFR and high C‐peptide level), liver damage (high levels of bilirubin and aspartate aminotransferase), or glucose metabolism (insulin), which were associated with plasma Aβ40 and Aß42 levels. These results suggest that plasma Aβ40 and Aß42 levels are susceptible to peripheral production and clearance and also that the Aβ40/42 ratio may reduce the effects of peripheral metabolism. After Aβ is released into the bloodstream, it undergoes clearance by multiple pathways, including degradation and phagocytosis by macrophages and neutrophils,^32^, ^33^ low‐density lipoprotein receptor‐related protein 1‐dependent metabolism by the liver,^34^ and excretion into urine.^35^ Peripheral Aβ production is dependent on insulin in the pancreas, adipose tissue, skeletal muscle, and liver.^36^ These basic mechanisms of peripheral Aß clearance warrant further study. On the contrary, the results of the correlation analysis revealed that the multiple correlation coefficient of eGFR surpassed that of the other variables, which is consistent with previous findings suggesting a correlation between renal function and plasma Aβ levels. Therefore, the incorporation of renal function into the mixed‐effects model was considered to be reasonable.

There are several limitations that need to be addressed. The relationship between change points and brain Aβ accumulation was not investigated because amyloid positron emission tomography findings were not available. A validation cohort that performs amyloid PET or spinal fluid testing when the rate of plasma Aβ changes exceeds +2SD is needed. Furthermore, since cohort participants were recruited from a population of Iwaki area residents on a voluntary basis, there may have been a self‐selection bias due to the relatively young age demographic of the study sample. Moreover, the large sample size in the present study allowed us to incorporate numerous explanatory variables into the mixed‐effects model, which was a significant advantage; however, it may also concomitantly lead to an overestimation of the relationship between blood collection data and plasma Aβ due to oversampling. Another limitation is that our cohort did not include many mild cognitive impairment and AD participants and not all subjects were followed up for 4 years. To overcome this issue, we adopted a mixed‐effects model. Nevertheless, the present results provide a more detailed understanding of the extent to which this measurement error may occur.

## Conflicts of Interest

None of the authors have any conflicts of interest to report.

## Author Contributions

Takumi Nakamura, Takeshi Kawarabayashi, Shigeyuki Nakaji, and Mikio Shoji conceptualized and designed the study. Takumi Nakamura, Takeshi Kawarabayashi, Nakahata Naoko, Ken Itoh, Kazushige Ihara, Shigeyuki Nakaji, and Mikio Shoji acquired and analyzed data. Takumi Nakamura, Takeshi Kawarabayashi, Ken Itoh, Kazushige Ihara, Shigeyuki Nakaji, Yoshio Ikeda, Masamitsu Takatama, and Mikio Shoji drafted the text and prepared the figures.
